# Sex Differences in Elderly Multiple Sclerosis Patients Undergoing Neurorehabilitation: How Many Things are Taken for Granted? A Retrospective Study

**DOI:** 10.1177/08919887251354899

**Published:** 2025-07-31

**Authors:** Davide Cardile, Maria Grazia Maggio, Lilla Bonanno, Mirjam Bonanno, Rosaria De Luca, Francesco Corallo, Fausto Famà, Amelia Rizzo, Angelo Quartarone, Rocco Salvatore Calabrò

**Affiliations:** 1 Neurorehabilitation Unit 120349 , IRCCS Centro Neurolesi Bonino Pulejo, Messina, Italy; 2 Department of Clinical and Experimental Medicine 18980 , University of Messina, Messina, Italy

**Keywords:** gender differences, Lokomat, multiple sclerosis, neurorehabilitation, virtual reality

## Abstract

**Background:**

Our aim is to evaluate the intricate dynamics of gender differences in cognitive rehabilitation outcomes among older adults with MS undergoing cognitive rehabilitation using robotics plus virtual reality.

**Methods:**

This retrospective study included 80 RRMS patients aged >60 years, matched for demographic and clinical variables and divided into two groups. The experimental group (EG, n = 40) received Lokomat Free-D training with VR integration, while the control group (CG, n = 40) underwent traditional rehabilitation. Cognitive, functional, and emotional outcomes were assessed before and after treatment.

**Results:**

Intergroup analysis revealed significantly greater improvements in the EG compared to the CG in MoCA (*P* < 0.001 in males, *P* = 0.001 in females), FIM (*P* = 0.02 in females), and HRS-A (*P* = 0.01 in males, *P* = 0.005 in females). Within-group analyses showed that both males and females in the EG experienced significant improvements across all domains (*P* < 0.001), but in the CG, improvements were more limited, particularly in mood scores. Notably, a positive correlation between MoCA and gender was found in EG (r = 0.47; *P* = 0.002), suggesting stronger cognitive gains among women.

**Conclusion:**

Our results provide preliminary data on the influence of gender differences on neurological rehabilitation outcomes, which should be evaluated and taken into due consideration to personalize and improve rehabilitation treatment.

## Introduction

Multiple sclerosis (MS) is a chronic immune-mediated disease of the central nervous system, characterized by inflammation, demyelination, and neurodegeneration. It presents a wide range of neurological symptoms that vary in type, intensity, and progression, often resulting in cognitive decline and physical disability over time.^1,2^ Although MS typically affects young adults, with a peak incidence between 30 and 35 years of age, advances in disease-modifying therapies have led to an increase in life expectancy, increasing the number of older adults living with MS.^1^ In this population, the cumulative effects of aging and disease-related damage can exacerbate functional and cognitive deficits. Therefore, MS represents a particular challenge in older adults, as the interplay between aging and neurodegeneration can further compromise quality of life and functional independence.^1,2^ In this context, gender may also play a crucial role in shaping rehabilitation outcomes, due to both biological and psychological differences. Indeed, sex-related variations in neuroinflammatory responses, hormonal regulation, and neuroplasticity may influence how patients respond to treatment. Additionally, differences in coping strategies, emotional involvement, and motivation could modulate engagement with and benefit from therapy. These aspects underscore the need for gender-sensitive, age-appropriate rehabilitation approaches aimed at optimizing outcomes in elderly individuals with MS.^3-5^

Therefore, the development of personalized approaches tailored to individual characteristics (ie, age, cognitive and emotional profile, gender) is now considered a priority in neurorehabilitation.^5^

Although the prevalence of MS is higher in older women (with a female to-male ratio is around 2.3-3.5:1), research often focuses on male-centered clinical and preclinical studies.^3,4^ Despite the well-documented benefits of individualized treatment strategies, the specific contribution of gender to rehabilitation outcomes in elderly patients with MS remains poorly investigated. However, investigating gender differences in rehabilitation outcomes presents several challenges. Many clinical trials are not adequately powered to conduct subgroup analyses by gender, and sample sizes in older MS populations are often limited. Furthermore, sex is frequently treated as a covariate rather than a variable of primary interest, reducing the interpretability of gender-specific effects. These limitations contribute to the current research gap and highlight the need for focused studies addressing sex-related differences in rehabilitation outcomes. It is important to highlight that several studies have demonstrated significant differences in the course of the disease related to biological sex.^5-8^ In particular, it has been observed that women have an earlier onset of the disease, may experience greater relapses, and tend to manifest the form of MS later in the course of the disease.^5-7^ Additionally, women may experience slower disability progression compared to men.^8^ These differences suggest a potential role of biological sex in shaping disease trajectory and response to treatment, further emphasizing the need for personalized rehabilitation strategies. Recent studies highlighted the potential role of sex hormones, particularly progesterone and estrogen, in ameliorating secondary brain injury, suggesting a broader neurobiological context for gender differences in rehabilitation outcomes.^9-12^ Neuron derived 17 β-estradiol (E2) and astrocyte-derived E2 emerge as key players, influencing synaptic plasticity, memory, and neuroprotection, potentially explaining observed cognitive differences between sex.^13^

Psychological factors also play a crucial role in rehabilitation response. Studies have consistently identified higher rates of depression and anxiety among female MS patients, potentially impacting motivation, adherence, and cognitive performance during rehabilitation. A previous study from our group^10^ has reinforced the evidence on the complex interplay between gender, anxiety, and stress in MS, particularly among young female patients.^11^

Another important factor influencing rehabilitation efficacy is exercise. Emerging research suggests that gender may moderate the relationship between physical activity and cognitive improvement in MS patients.^14^ It has been found that older women showed greater enhancements in attention and executive functions following structured physical training, reinforcing the importance of individualized rehabilitation approaches.^14^ Despite these insights, few clinical trials have conducted sex-specific subgroup analyses to assess the effectiveness of rehabilitation treatments.^9-15^ Furthermore, a meta-analysis revealed no distinct sex-based differences in rehabilitation outcomes, underscoring the need for further investigations on this topic.^15^

Given the limited research on gender differences in rehabilitation outcomes among older MS patients, our study aims to explore the impact of sex on cognitive and functional recovery following neurorehabilitation. Specifically, we evaluate how an innovative rehabilitation approach, integrating robotics and virtual reality (VR), may differentially affect men and women with MS. We hypothesize that gender influences rehabilitation outcomes, particularly in cognitive and emotional domains, and that VR-based interventions may offer specific benefits compared to traditional rehabilitation approaches.

## Material and Methods

### Study Design and Population

This retrospective study analyzed data from patients diagnosed with Relapsing-Remitting Multiple Sclerosis (RRMS) who attended the Robotic and Behavioral Neurorehabilitation Service at IRCCS Centro Neurolesi Bonino-Pulejo (Messina, Italy) between February 2018 and March 2020. Data were retrieved from the institution’s electronic medical records.

This study was conducted and reported in accordance with the Strengthening the Reporting of Observational Studies in Epidemiology (STROBE) guidelines and followed the principles of the Declaration of Helsinki. The medical records of 310 patients with RRMS treated in our unit were reviewed.

The inclusion criteria were: (i) diagnosis of RRMS according to the McDonald review criteria^16^; (ii) stable therapy of the patient for at least six months before entry into the study; (iii) absence of severe cognitive impairment (Montreal Cognitive Assessment - MoCA< 15)^17^; (iv) age >60 years.

The exclusion criteria were: (i) presence of serious medical and psychiatric diseases that could interfere with the Innovative Treatment (IT); (ii) clinical and/or neuroradiological relapse of MS in the 6 months before the enrolment; (iii) use of steroids or psychoactive drugs in the last six months; (iv) Modified Ashworth Scale >3; (v) Expanded Disability Status Scale (EDSS) > 7^18^; (vi) Severe musculoskeletal conditions preventing adaptation to the Lokomat Free-D; (vii) Lack of cooperation, aggressive behavior, transient psychosis, or severe cognitive impairment.

### Procedures

Patients were assigned to two rehabilitation approaches based on clinical eligibility criteria and physician recommendations.^19^ The two groups were balanced for age, sex, and disability level (EDSS) to minimize selection bias. The experimental group (EG) underwent Lokomat Free-D-assisted training with integrated virtual reality (VR), while the control group (CG) received traditional rehabilitation.

The traditional motor rehabilitation was standardized across all patients and based on the internal clinical protocols of the rehabilitation unit. It consisted of structured sessions including balance training, stretching, muscle strengthening, and gait re-education. Cognitive stimulation included standardized paper-and-pencil tasks targeting attention and executive functions, such as visual search, sequence linking, and element categorization. All sessions followed a consistent structure, with intensity and progression predefined according to clinical guidelines. Treatments were delivered by experienced therapists trained in the application of these standardized procedures, with only individual adaptations based on patients’ baseline abilities.

In the EG, the Lokomat Free-D was used in combination with a VR interface consisting of an avatar-based visual feedback system. The VR component was designed to enhance motor-cognitive integration and promote patient engagement.^1,17-22^ During gait training, patients controlled a 2D avatar displayed on a screen in front of them, which mirrored their walking patterns in real time. This feedback created an interactive environment requiring patients to focus on task-related visual cues and maintain coordination between movement and avatar response. Tasks included navigating through virtual pathways, avoiding obstacles, and following specific visual targets, all of which were aimed at stimulating sustained attention and executive functions. The immersive and responsive nature of the system encouraged high levels of active participation, increasing motivation, and cognitive involvement during motor rehabilitation. While the primary goal of the Lokomat Free-D intervention was motor rehabilitation, the integration of a VR interface aimed to stimulate cognitive domains, particularly attention and executive functions, through motor-cognitive interaction and engagement.

For each group, the rehabilitation protocol included a total of 40 training sessions (five sessions per week for eight weeks, following previous standard and clinical research protocols) lasting approximately one hour each for both groups. All patients underwent clinical and neuropsychological evaluations at the beginning (T0) and at the end (T1) of the rehabilitation program.

### Outcomes Measures

Upon admission, all patients underwent assessments of both motor and cognitive functions by the multidisciplinary team (neurologist, physiatrist, psychologist, physiotherapist, and nurse).^20,21^ The clinical assessment battery included the Montreal Cognitive Assessment (MoCA) to assess general cognitive functioning; the Functional Independence Measure (FIM) to evaluate the patient’s level of disability and response to interventions; the Hamilton Anxiety Inventory (HRS-A) for mood assessment; Goal Attainment Scaling (GAS) to evaluate the achievement of therapeutic goals.

### Statistical Analysis

A priori sample size estimation was performed using G*Power 3.1.9.7, based on a Wilcoxon-Mann-Whitney test for independent groups. An effect size of 0.7 (moderate-large), α = 0.05, and power = 0.80 were considered. The results suggested a minimum sample size of 35 participants per group (total: 70). Given the retrospective nature of the study, we reviewed the medical records of 310 patients with RRMS treated in our unit. From this larger cohort, 40 patients per group (total: 80) were selected to meet the sample size requirement and ensure adequate statistical power. Participants were assigned to the experimental or control group based on clinical eligibility and treatment history. To reduce selection bias, patients were matched for age, sex, and disability level. In particular, EDSS scores were matched within a tolerance range of ±0.4 to ensure a comparability of disability levels between groups.

The analyses were conducted using the open-source R4.2.2 software package provided by the R Foundation for Statistical Computing, Vienna, Austria. A confidence level of 95% was established with a 5% alpha error. Statistical significance was determined at a *P*-value of less than 0.05.

The analysis was carried out using descriptive statistics to examine the sociodemographic characteristics of respondents, followed by calculating the mean and standard deviation for two groups. The normality of the data distribution was assessed using the Shapiro-Wilk test. Correlations among variables were determined using Spearman’s coefficient, or the point of biserial correlation coefficient when one of the variables was dichotomous. We performed an interaction effect analysis (improved time) by calculating the T1–T0 differences in clinical variables scores. Qualitative variables were expressed in numbers and percentages, and the Chi-square (χ2) test was utilized for their comparison. For non-normally distributed data, the analysis employed either the Mann–Whitney U test or the Wilcoxon signed-rank test, depending on what was considered appropriate. Effect sizes (Cohen’s d or rank-biserial correlation r) were calculated to estimate the magnitude of differences between groups. When applicable, 95% confidence intervals were also reported to enhance interpretability. Given the exploratory nature of the study and the relatively small sample size, no correction for multiple comparisons was applied. The potential increase in the risk of false positives is acknowledged and discussed as a limitation.

## Results

**Figure 1.**
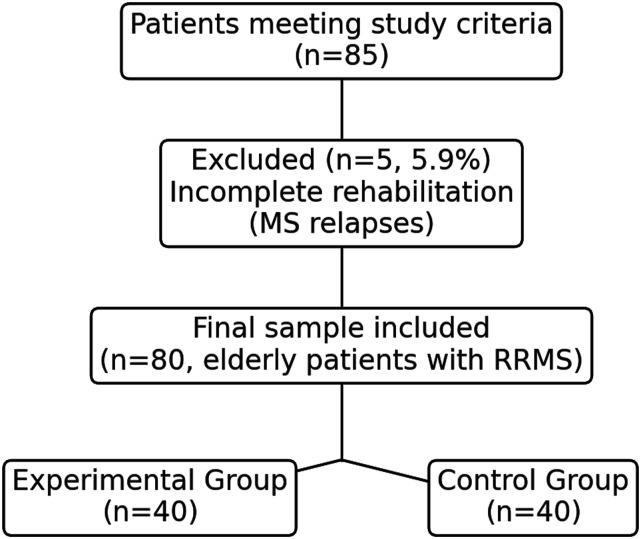
Patient selection flow diagram.

Initially, 85 patients met the study criteria, but 5 (5.9%) were excluded due to incomplete rehabilitation caused by adverse events (MS relapses). Therefore, the final sample included 80 elderly patients with RRMS, divided into two groups: the experimental group (EG, n = 40) and the control group (CG, n = 40) (Figure 1). Baseline clinical characteristics were comparable between groups. The mean EDSS score across all participants was 3.42 ± 0.73, indicating a moderate level of disability. No significant differences in EDSS scores were observed between the experimental and control groups at baseline. The socio-demographic characteristics of the two groups are shown in Table 1.

**Table 1.** Socio-Demographic and Characteristics of Groups.

	Experimental group				Control group				1.1 ∼ 2.1	1.2 ∼ 2.2
	All	Male (1.1)	Female (1.2)	*P*	All	Male (2.1)	Female (2.2)	*P*	*P*	*P*
N. subject	**40**	20	20		**40**	20	20			
Age	**69.73 ± 4.04**	69.35 ± 4.48	70.10 ± 3.63	0.80	**67.08 ± 4.98**	65.45 ± 3.12	68.70 ± 5.97	0.09	0.004^*^	0.34
Education	**13.0 (8.0-13.0)**	13.0 (8.0-13.0)	13.0 (8.0-13.0)	0.86	**13.0 (8.0-13.0)**	13.0 (8.0-13.0)	13.0 (8.0-13.0)	0.41	0.74	0.47
**Disease duration**	**37.65 ± 8.60**	**38.3 ± 8.06**	**37.0 ± 9.28**		**32.65 ± 11.04)**	**31.35 ± 11.35**	**33.95 ± 10.84**			
**EDSS**	**3.54 ± 0.63**	**3.43 ± 0.56**	**6.36 ± 0.69**	**0.32**	**3.47 ± 0.68**	**3.59 ± 0.73**	**3.34 ± 0.61**	**0.25**	**0.44**	**0.17**

Data are presented as means ± standard deviations for normally distributed variables and medians (first and third quartiles) for non-normally distributed variables. In bold are the *P*-values obtained from the nonparametric test (Mann-Whitney U or Wilcoxon signed rank test for intra-inter group analysis respectively).

Gender-based analysis showed similar characteristics for the two groups, but no significant differences emerged (*P* = 0.07). Intra-group analysis Spearman’s correlation highlighted a positive significant correlation between MOCA and gender (r = 0.47; *P* = 0.002) in EG and no significant correlations were found in CG. In EG, we found significant differences in intra-gender in all neuropsychological scores (*P* < 0.001) while in inter-gender we showed a significant difference in GAS at T0 (*P* = 0.03) (Table 2 and Table S1). In CG, we highlighted significant differences in FIM (*P* < 0.001), MOCA (*P* = 0.002), and GAS (*P* < 0.001) but not in HRS-A (*P* = 0.12) for the male group, and IM (*P* < 0.001), MOCA (*P* = 0.02) and GAS (*P* < 0.001) but no in HRS-A (*P* = 0.17) for female group (Table 2 and Table S1). No highlighted no significant correlations within gender (males and females) in the two groups (EG and CG) (Table S2).

**Table 2.** Comparison of Functional, Cognitive, and Psychological Outcomes Between Experimental and Control Groups.

	Experimental group			Control group			1.1 ∼ 2.1	1.2 ∼ 2.2
	Male (1.1)	Female (1.2)	*P*	Male (2.1)	Female (2.2)	*P*	*P*	*P*
FIM T0	70.60 ± 15.74	72.0 (58.0-85.2)	0.71	76.80 ± 19.87	78.85 ± 21.18	0.75	0.55	0.07
FIM T1	102.90 ± 9.98	98.75 ± 12.49	0.31	94.0 ± 18.94	91.0 (83.5-96.5)	0.51	0.07	**0.02** ^a^
*P*	**<0.001** ^a^	**<0.001** ^a^		**<0.001** ^a^	**<0.001** ^a^			
MOCA T0	22.75 ± 1.74	22.60 ± 1.43	0.98	21.95 ± 2.54	22.55 ± 3.12	0.51	0.51	0.95
MOCA T1	25.5 (25.0-26.0)	26.0 (26.0-27.0)	0.07	22.65 ± 2.48	23.30 ± 3.06	0.46	**<0.001** ^a^	**0.001** ^a^
*P*	**<0.001** ^a^	**<0.001** ^a^		**0.002** ^a^	**0.02** ^a^			
HRS-A T0	9.90 ± 3.67	9.85 ± 2.62	0.96	7.40 ± 4.04	8.10 ± 3.29	0.51	0.03^a^	0.07
HRS-A T1	4.0 (2.75-4.25)	4.0 (3.0-5.2)	0.58	6.95 ± 3.87	6.0 (6.0-11.2)	0.73	**0.01** ^a^	**0.005** ^a^
*P*	**<0.001** ^a^	**<0.001** ^a^		0.12	**0.17**			
GAS T0	37.93 ± 1.38	36.51 ± 3.51	0.10	37.6 (37.6-37.7)	37.6 (37.6-37.79)	0.55	0.54	**0.004** ^a^
GAS T1	67.20 (61.95-68.6)	67.2 (60.48-68.6)	0.88	67.2 (60.8-68.6)	66.75 (55-9-68.6)	0.80	0.17	**0.71**
*P*	**<0.001** ^a^	**<0.001** ^a^		**<0.001** ^a^	**<0.001** ^a^			

^a^Legend: Expanded Disability Status Scale (EDSS); Functional Independence Measure (FIM); Montreal Cognitive Assessment (MoCA); Goal Attainment Scaling (GAS); Hamilton Anxiety Inventory (HRS-A). Data are presented as means ± standard deviations for normally distributed variables and medians (first and third quartiles) for non-normally distributed variables. In bold are the *P*-values obtained from the nonparametric test (Mann-Whitney U or Wilcoxon signed rank test for intra-inter group analysis respectively).

**Figure 2.**
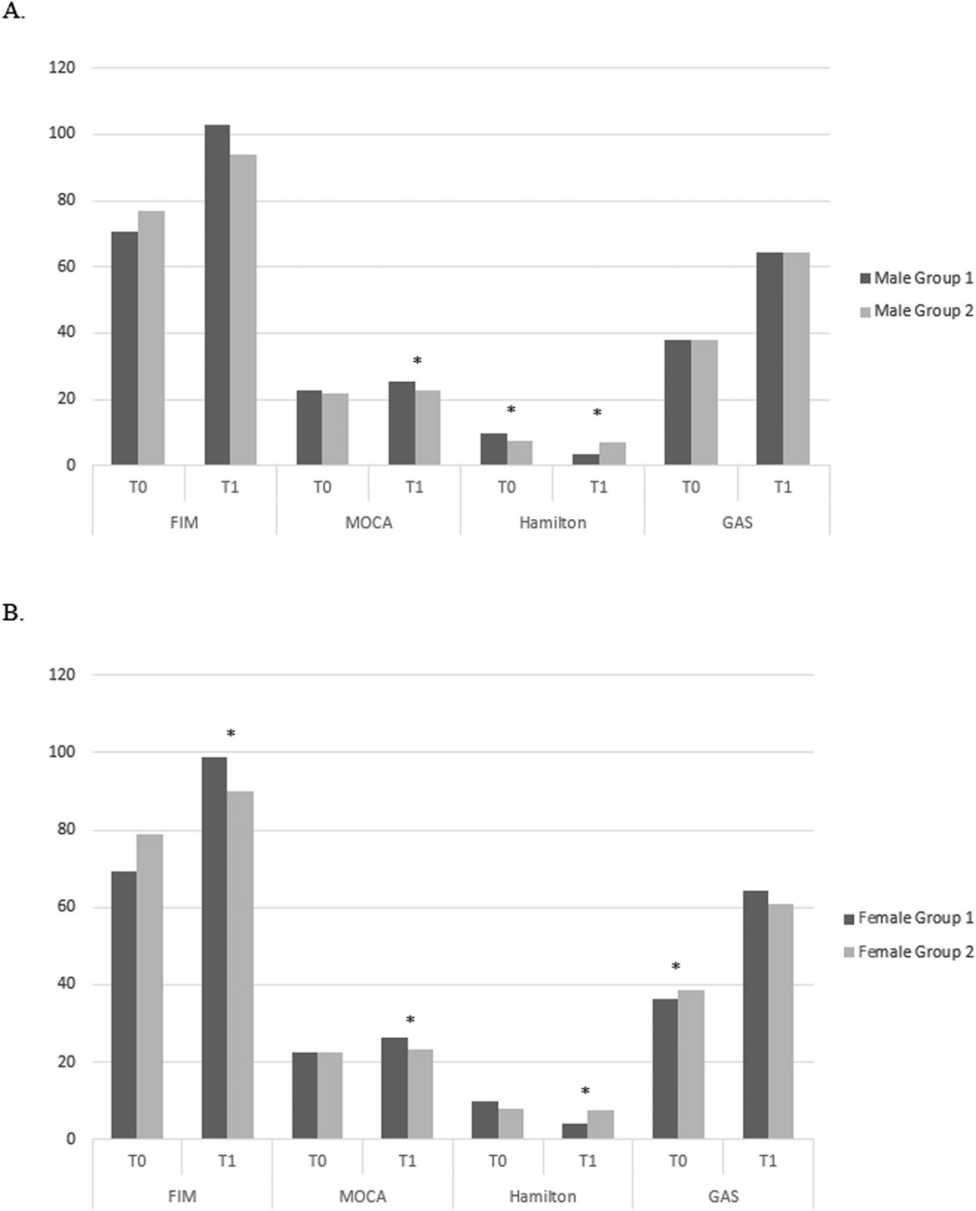
Inter-group analysis. (A) Differences in neuropsychological score between groups in males at baseline and T1. (B) Differences in neuropsychological score between groups in females in baseline and T1. *P < 0.05.

Inter-group analysis (Figure 2), male EG vs male CG, showed significant differences in MOCA at T1 (*P* < 0.001), HRS-A at T0 (*P* = 0.03) and T1 (*P* = 0.01) (Table 3 and Table S3), while, female EG vs female CG showed significant differences in FIM at T1 (*P* = 0.02), MOCA at T1 (*P* = 0.001), HRS-A at T1 (*P* = 0.005) and GAS at T0 (*P* = 0.004) (Table 4 and Table S3).

**Table 3.** Comparison of Functional, Cognitive and Psychological Outcomes Between Males and Groups.

	Male EG (n = 20)	Male CG (n = 20)	*P*-value
FIM T0	70.60 ± 15.74	76.80 ± 19.87	0.55
FIM T1	102.90 ± 9.98	94.0 ± 18.94	0.07
MOCA T0	22.75 ± 1.74	21.95 ± 2.54	0.51
MOCA T1	25.5 (25.0-26.0)	22.65 ± 2.48	<0.001*
HRS-A T0	9.90 ± 3.67	7.40 ± 4.04	0.03*
HRS-A T1	4.0 (2.75-4.25)	6.95 ± 3.87	0.01*
GAS T0	37.93 ± 1.38	37.6 (37.6-37.7)	0.54
GAS T1	67.20 (61.95-68.6)	67.2 (60.8-68.6)	0.99

Legend: EG = Experimental Group; CG = Control Group; Expanded Disability Status Scale (EDSS); Functional Independence Measure (FIM); Montreal Cognitive Assessment (MoCA); Goal Attainment Scaling (GAS); Hamilton anxiety Inventory (HRS-A). Data are presented as means ± standard deviations for normally distributed variables and medians (first and third quartiles) for non-normally distributed variables. In bold are the *P*-values obtained from the nonparametric test (Mann-Whitney U or Wilcoxon signed rank test for intra-inter group analysis respectively).

*P < 0.05.

**Table 4.** Comparison of Functional, Cognitive and Psychological Outcomes Between Females and Groups.

	Female EG (n = 20)	Female CG (n = 20)	*P*-value
FIM T0	72.0 (58.0-85.2)	78.85 ± 21.18	0.07
FIM T1	98.75 ± 12.49	91.0 (83.5-96.5)	0.02*
MOCA T0	22.60 ± 1.43	22.55 ± 3.12	0.95
MOCA T1	26.0 (26.0-27.0)	23.30 ± 3.06	0.001*
HRS-A T0	9.85 ± 2.62	8.10 ± 3.29	0.07
HRS-A T1	4.0 (3.0-5.2)	6.0 (6.0-11.2)	0.005*
GAS T0	36.51 ± 3.51	37.6 (37.6-37.79)	0.004*
GAS T1	67.2 (60.5-68.6)	66.75 (55.9-68.6)	0.71

Legend: EG = Experimental Group; CG = Control Group; Expanded Disability Status Scale (EDSS); Functional Independence Measure (FIM); Montreal Cognitive Assessment (MoCA); Goal Attainment Scaling (GAS); Hamilton anxiety Inventory (HRS-A). Data are presented as means ± standard deviations for normally distributed variables and medians (first and third quartiles) for non-normally distributed variables. In bold are the *P*-values obtained from the nonparametric test (Mann-Whitney U or Wilcoxon signed rank test for intra-inter group analysis respectively).

*P < 0.05.

## Discussion

To the best of our knowledge, this retrospective study provides novel insights into the impact of gender differences on the outcomes of intensive neurorehabilitation in elderly patients with RRMS. Our findings demonstrate that robot-assisted rehabilitation integrated with VR leads to greater improvements in both cognitive and functional domains compared to traditional rehabilitation approaches. All participants (male and female) in the EG showed a significant improvement in all the areas considered by the evaluation battery (FIM, MOCA, HRS-A, GAS). On the contrary, in CG this improvement was significant for the FIM, MOCA and GAS scales, but no improvement was observed in HRS-A. Therefore, IT would appear to be more effective than traditional rehabilitation in improving disability, cognitive, and mood-related aspects. Notably, we have observed that our patients have the perception of having achieved their set goals (GAS). Furthermore, our results highlight the high usability of Lokomat Free D plus VR, particularly among patients with higher levels of disability, as observed in other neurological diseases.^21-24^ Although not directly measured in our study, previous research suggests that the perceived interactivity and technological novelty of such systems may foster patient motivation and engagement, potentially enhancing compliance and psychological outcomes such as self-esteem and sense of agency.^25-27^ From a psychological point of view, interfacing with cutting-edge robotic equipment could make the patient feel more involved in the therapeutic process, as an active subject, with an increase in empowerment.^28-30^ According to our findings, various authors show that rehabilitation approaches, both traditional and innovative, could improve the rehabilitation outcome, perceived disability, and performance in gait and balance.^24,31-33^ In particular, it was highlighted that IT could allow improvements greater than traditional ones both in functional outcomes and in mood,^1,10^ as observed in our overall sample, especially in women. Although the mechanisms are still being studied, IT, using robotics and virtual reality, has been shown to have an impact on a neurophysiological level.^34^ Firstly, this approach boosts the connectivity between different functional areas, with positive repercussions at the level of the cortical representation of the muscles of the affected limb.^27,35-37^ It would also appear to increase the activity of frontal regions^38,39^ and improve neural plasticity, including the maladaptive one.^40,41^ Another interesting aspect of IT in our study is related to the differences in results by gender. As mentioned, IT was more effective than traditional methods, but especially in women. Although both genders had good outcomes, women presented greater benefits from innovative rehabilitation (robotics with VR) in cognitive and emotional aspects compared to conventional rehabilitation. These results are in line with a study from our group relating to patients suffering from traumatic brain injury. In fact, we found improvements in cognitive global functioning, and reduced disability and anxiety levels in women compared to men following an IT performed with VR.^11^ In line with these findings, Ji et al, after having analyzed health data from 400,000 US adults from 1997 to 2017, found that for the same number of hours of exercise, women achieved greater health benefits than men.^42^ Various biological mechanisms may contribute to gender differences observed in rehabilitation outcomes. In particular, sex-related differences in neuroplasticity and hormonal modulation of the central nervous system have been hypothesized to play a role.^43^ Some studies suggest that females may exhibit higher neuroplastic potential in specific brain regions, such as the hippocampus and prefrontal cortex, potentially enhancing responsiveness to cognitive and emotional rehabilitation.^44-46^ Furthermore, sex hormones, including estrogen and testosterone, influence synaptic plasticity, neurogenesis, and neurotransmitter systems, thereby modulating the brain’s capacity to adapt to training and external stimuli.^47-50^ These factors, although not measured directly in our study, might partly explain the greater benefits observed in female participants.^47,48,51^ Therefore, considering these biological factors is essential to tailor rehabilitation strategies to individual needs, ultimately optimizing outcomes for patients undergoing cognitive rehabilitation, particularly in conditions such as MS.

On the other hand, another relevant finding is that both genders report improvements in mood after IT, which would therefore allow a reduction in symptoms with positive effects on training, therapeutic alliance, and compliance. This result aligns with a growing body of literature highlighting the benefits of integrating advanced technologies into rehabilitation to improve mood and overall psychological well-being. For example, a meta-analysis by Laver et al. demonstrated that VR interventions significantly reduced depressive symptoms and improved mood in various patient populations, including men and women.^52^ The interactive and immersive nature of VR contributes to a more engaging rehabilitation experience, promoting greater patient motivation and satisfaction. For example, Gao et al found that patients engaging in IT based rehabilitation experienced substantial reductions in anxiety and depressive symptoms, with positive effects noted in both male and female participants. However, gender differences in the impact of VR on mood have been reported in various studies.^53^ Jingili et al. found that women tend to experience more significant improvements in mood and emotional well-being after virtual reality rehabilitation than men.^54^ This may be due to women’s generally higher engagement and responsiveness to the immersive and interactive elements of VR, which can enhance their overall emotional experience. On the other hand, although men also benefit from VR-based interventions, mood improvements are often less pronounced than their female counterparts. This is in line with the findings of Peng et al who observed that both genders experienced improvements in mood thanks to virtual reality, but women showed more substantial improvements,^55^ as in our sample. Additionally, traditional rehabilitation methods often fail to address the psychological aspects of recovery. Georgiev et al. highlighted traditional approaches lacking the interactive elements of IT, may not fully engage patients, leading to less significant improvements in mood.^56^ This is particularly relevant when considering gender differences, as women may experience higher levels of psychological distress and benefit more from the immersive and interactive nature of virtual reality than men. According to these observations, our findings showed that both men and women report improvements in mood with IT and VR. Notably, women experience greater benefits, which is in line with literature suggesting that women are often more responsive to the immersive and immersive features of VR and IT. By integrating IT and VR into rehabilitation practices, it may be possible to better address gender-specific needs and optimize therapeutic outcomes for all patients. This approach not only enhances functional improvements but also supports a more holistic rehabilitation strategy that takes psychological well-being into account.

One of the strengths of this work is the emphasis on gender difference and its impact on neurorehabilitation. Although gender differences are significant on several levels in rehabilitation processes, this issue is often overlooked in literature and especially in clinical practice. We showed that awareness of gender differences in response to treatment could encourage the personalization of treatment, favoring better rehabilitation outcomes.

Although the retrospective design of this study and the use of data extracted from electronic medical records helped minimize assessment errors, this study has some limitations, including the small sample and lack of follow-up. It would be very interesting to see in a larger sample if and how the different IT influences the variables considered in this study over time. Furthermore, it would be very interesting to consider how age, sex, environmental factors, fatigue, lifestyle, experiences, and different health needs can influence the response to treatment and consequently the therapeutic outcomes. Due to the retrospective nature of the study, some potentially relevant clinical variables, such as the type of Disease Modifying Therapies, were not consistently available in in-patient records and could not be reliably included in the analyses. Another limitation is the exclusive use of the MoCA as a cognitive measure, which, although widely used, may not fully capture the spectrum of cognitive deficits typically observed in MS patients. Moreover, the selection of clinical tools was limited to those routinely administered and recorded in patient files, which may have restricted the breadth of neuropsychological and mood assessments. Additionally, we cannot exclude the influence of unmeasured confounding factors such as baseline cognitive reserve or previous cognitive training or socioeconomic differences, which may differ by gender and potentially impact rehabilitation outcomes. Future studies should address all these aspects through systematic data collection, and incorporate more variables to better isolate the effect of gender per se. A limitation of this study is the absence of multiple comparison corrections, which may have increased the likelihood of Type I errors. This choice was made due to the exploratory nature of the analysis and the limited sample size. Future studies should apply appropriate correction methods to validate these preliminary findings.

In conclusion, our findings represent exploratory, retrospective evidence on the potential influence of gender differences on neurological rehabilitation outcomes, which should be evaluated and taken into due consideration to personalize and improve rehabilitation treatment. These preliminary observations require confirmation in future prospective studies with larger, more diverse samples and controlled methodologies, to draw definitive conclusions. However, the differences between men and women regarding autoimmune diseases could not only be represented by the different prevalence but could also been described in the severity of symptoms, the course of the disease, the response to therapy, and survival. A patient-centric and gender-centric perspective could allow a more effective approach to the patient and his/her pathologies.

## Supplemental Material

**Supplemental Material -** Sex Differences in Elderly Multiple Sclerosis Patients Undergoing Neurorehabilitation: How Many Things are Taken for Granted? A Retrospective Study Supplemental Material for Sex Differences in Elderly Multiple Sclerosis Patients Undergoing Neurorehabilitation: How Many Things are Taken for Granted? A Retrospective Study by Davide Cardile, Maria Grazia Maggio, Lilla Bonanno, Mirjam Bonanno, Rosaria De Luca, Francesco Corallo, Fausto Famà, Amelia Rizzo, Angelo Quartarone, and Rocco Salvatore Calabrò in Journal of Geriatric Psychiatry and Neurology.

## Data Availability

The data associated with the paper are not publicly available but are available from the corresponding author on reasonable request.*
