# Supplementary material for: Sex Differences in Elderly Multiple Sclerosis Patients Undergoing Neurorehabilitation: How Many Things are Taken for Granted? A Retrospective Study
Source: J Geriatr Psychiatry Neurol. 2025 Jul 31;39(2):196–206. doi: 10.1177/08919887251354899 (PMC12799802; doi:10.1177/08919887251354899)
Supplement: Supplemental Material - Sex Differences in Elderly Multiple Sclerosis Patients Undergoing Neurorehabilitation: How Many Things are Taken for Granted? A Retrospective Study [file sj-pdf-1-jgp-10.1177_08919887251354899.pdf]

Table S1. Within-group comparisons (pre- vs post-intervention) for the Experimental and Control groups, stratified by sex. For each variable, the type of statistical test, test statistic, p-value, and effect size are reported. Depending on the distribution of the data, either Wilcoxon signed-rank tests or paired-sample t-tests were applied.

Group	Sex	Variable	Test	Statistic	p-value	Effect Size	95% CI (lower;upper)
Experimental	Male	FIM (T0-T1)	Wilcoxon	W00	<0.001	r=0.83	-
		MOCA (T0-T1)	Wilcoxon	W=0	<0.001	r=0.85	-
		Hamilton-D (T0-T1)	Wilcoxon	W=171	<0.001	r=0.79	-
		GAS (T0-T1)	Wilcoxon	W=0	<0.001	r=0.84	-
Experimental	Female	FIM (T0-T1)	Wilcoxon	W=0	<0.001	r=0.83	-
		MOCA (T0-T1)	Wilcoxon	W=0	<0.001	r=0.85	-
		Hamilton-D (T0-T1)	Wilcoxon	W=208	<0.001	r=0.82	-
		GAS (T0-T1)	Wilcoxon	W=0	<0.001	r=0.84	-
Control	Male	FIM (T0-T1)	T-Student	T=-6.03	<0.001	d=-0.53	(-0.72;-0.34)
		MOCA (T0-T1)	T-Student	T=-2.59	0.02	d=-0.24	(-0.43;-0.05)
		Hamilton-D (T0-T1)	Wilcoxon	W=65	0.17	r=0.21	-
		GAS (T0-T1)	Wilcoxon	W=0	<0.001	r=0.84	-
Control	Female	FIM (T0-T1)	T-Student	T=-6.05	<0.001	d =-0.88	(-1.23;-0.53)
		MOCA (T0-T1)	T-Student	T=-3.62	0.002	d =-0.28	(-0.43;-0.12)
		Hamilton-D (T0-T1)	Wilcoxon	W=43	0.12	r=0.26	-
		GAS (T0-T1)	Wilcoxon	W=0	<0.001	r=0.84	-

Effect sizes are reported as *r*, calculated for non-parametric comparisons (Wilcoxon tests). The *r* effect size was calculated as $r = Z/\sqrt{N}$, where Z is the standardized test statistic and N is the total number of observations.
For paired-sample t-tests, the effect size is reported as Cohen's d, with corresponding 95% confidence

intervals. Cohen's d values can be interpreted as follows: 0.2 = small, 0.5 = medium, 0.8 = large effect.

Table S2. Between-sex comparisons (male vs female) within the Experimental and Control groups, conducted separately at baseline (T0) and post-intervention (T1). For each variable, the statistical test used, test statistic, p-value, effect size, and 95% confidence interval (when applicable) are reported. Depending on data distribution, independent samples t-tests or Mann–Whitney U tests were applied.

		**Test**	**Statistic**	**p-value**	**Effect Size**	**95% CI (lower;upper)**
Experimental Group at T0	Age	T-Student	T=0.58	0.56	d=0.18	(-0.46;0.82)
	Education	U-Mann Whitney	W=193.5	0.86	r=0.17	-
	Disease Duration	T-Student	T=-0.47	0.64	d=-0.15	(-0.79;0.49)
	EDSS	T-Student	T=1.01	0.32	d=0.32	(-0.32;0.96)
	FIM	U-Mann Whitney	W=186	0.71	r=0.09	-
	MOCA	U-Mann Whitney	W=198.5	0.98	r=0.32	-
	Hamilton-D	T-Student	T=-0.05	0.96	d=-0.01	(-0.65;0.62)
	GAS	T-Student	T=-1.69	0.10	d=-0.53	(-1.18;0.12)
Experimental Group at T1	FIM	U-Mann Whitney	W=162.5	0.31	r=0.08	-
	MOCA	U-Mann Whitney	W=264.5	0.07	r=0.23	-
	Hamilton-D	U-Mann Whitney	W=220.5	0.58	r=0.03	-
	GAS	U-Mann Whitney	W=206	0.88	r=0.18	-
Control Group at T0	Age	T-Student	T=2.16	0.03	d=0.68	(0.02;1.34)
	Education	U-Mann Whitney	W=229	0.41	r=0.03	-
	Disease Duration	T-Student	T=0.74	0.46	d=0.23	(-0.41;0.88)
	EDSS	T-Student	T=-1.17	0.25	d=-0.37	(-1.02;0.27)
	FIM	T-Student	T=0.31	0.75	d=0.10	(-0.54;0.74)
	MOCA	U-Mann Whitney	W=0.67	0.51	r=0.21	(-0.43;0.85)
	Hamilton-D	U-Mann Whitney	W=224.5	0.51	r=0.005	-
	GAS	U-Mann Whitney	W=220	0.55	r=0.02	-
Control Group at T1	FIM	T-Student	T=-0.67	0.51	d=-0.21	(-0.85;0.43)
	MOCA	T-Student	0.74	0.46	d=0.23	(-0.41;0.87)
	Hamilton-D	U-Mann Whitney	W=213	0.73	r=0.10	-
	GAS	U-Mann Whitney	W=190.5	0.80	r=0.13	-

Effect sizes are reported as *r*, calculated for non-parametric comparisons (Wilcoxon tests). The r effect size was calculated as $r = Z/\sqrt{N}$, where Z is the standardized test statistic and N is the total number of observations.

For parametric tests (independent samples t-test), effect size is reported as Cohen's d, with corresponding 95% confidence intervals.

Table S3. Between-group comparisons (Experimental vs Control Group) stratified by sex (male and female) and timepoint (T0 = baseline; T1 = post-intervention). For each variable, the statistical test used, test statistic, p-value, effect size, and 95% confidence interval (when applicable) are reported. Depending on the data distribution, independent-samples t-tests or Wilcoxon rank-sum tests (Mann–Whitney U) were applied.

		**Test**	**Statistic**	**p-value**	**Effect Size**	**95% CI (lower;upper)**
Experimental vs Control Group Male T0	Age	T-Student	T=3.20	0.003	d=1.01	(0.33;1.69)
	Education	U-Mann Whitney	W=212	0.74	r=0.10	-
	Disease Duration	T-Student	T=2.23	0.03	d=0.70	(0.05;1.36)
	EDSS	T-Student	T=-0.78	0.44	d=-0.25	(-0.89;0.39)
	FIM	U-Mann Whitney	U=177.5	0.55	r=0.02	-
	MOCA	U-Mann Whitney	U=224	0.51	r=0.006	-
	Hamilton-D	U-Mann Whitney	U=281.5	0.03	r=0.30	-
	GAS	U-Mann Whitney	U=180.5	0.54	r=0.01	-
Experimental vs Control Group Male T1	FIM	T-Student	T=0.06	0.95	d=0.02	(-0.62;0.66)
	MOCA	U-Mann Whitney	U=341	<0.001	r=0.59	-
	Hamilton-D	U-Mann Whitney	U=108	0.01	r=0.36	-
	GAS	U-Mann Whitney	U=199.5	0.99	r=Inf	-
Experimental vs Control Group Female T0	Age	T-Student	T=0.89	0.38	d=0.28	(-0.36;0.93)
	Education	U-Mann Whitney	W=174.2	0.47	r=0.01	-
	Disease Duration	T-Student	T=0.95	0.34	d=0.30	(-0.34;0.95)
	EDSS	T-Student	T=1.40	0.17	d=0.44	(-0.20;1.09)
	FIM	U-Mann Whitney	U=132.5	0.07	r=0.23	-
	MOCA	T-Student	T=0.06	0.95	d=0.02	(-0.62;0.66)
	Hamilton-D	T-Student	T=1.86	0.07	d=0.59	(-0.06;1.24)
	GAS	U-Mann Whitney	U=109.5	0.004	r=0.42	-
Experimental vs Control Group Female T1	FIM	U-Mann Whitney	U=282.5	0.03	r=0.31	-
	MOCA	U-Mann Whitney	U=0.45	0.001	r=0.45	-
	Hamilton-D	U-Mann Whitney	U=316	0.001	r=0.45	-
	GAS	U-Mann Whitney	U=213.5	0.71	r=0.09	-

Effect sizes are reported as r, calculated for non-parametric comparisons (Wilcoxon tests). The r effect size was calculated as $r = Z/\sqrt{N}$, where Z is the standardized test statistic and N is the total number of observations.

For parametric tests (independent-samples t-tests), effect size is reported as Cohen's d, with corresponding 95% confidence intervals.
